# Intestinal Reactive Amyloid A (AA) Amyloidosis in a Patient With Multiple Myeloma: A Case Report and Literature Review

**DOI:** 10.7759/cureus.42906

**Published:** 2023-08-03

**Authors:** Flor Rosado, Patxis Taveras, Vijay Gayam, Nithan Narendra, Ivette Vigoda

**Affiliations:** 1 Internal Medicine, St. Barnabas Hospital Health System, Bronx, USA; 2 Gastroenterology and Hepatology, The Brooklyn Hospital Center, Brooklyn, USA; 3 Gastroenterology, St. Barnabas Hospital Health System, Bronx, USA; 4 Hematology and Oncology, St. Barnabas Hospital Health System, Bronx, USA

**Keywords:** case report, aa amyloidosis, intestinal amyloidosis, gastrointestinal bleeding, multiple myeloma, amyloidosis

## Abstract

Amyloidosis is a rare group of disorders characterized by the extracellular deposition of misfolded protein aggregates that interfere with the function of the tissue affected. In some patients, the presenting symptom of monoclonal gammopathies, such as multiple myeloma, can be a gastrointestinal bleed with a further report of amyloidosis in gastrointestinal samples. In all the cases the pathology report is read as AL (light chain) amyloidosis. We present a case of a 57-year-old male patient with no medical history who debuted with gastrointestinal bleeding. A colonoscopy revealed a colonic ulcer with a pathologic diagnosis of amyloid A (AA) amyloidosis. Further investigation led to the finding of multiple myeloma (MM) with no evidence of systemic amyloidosis. Although there is little evidence in the literature of the association or even causative relationship between multiple myeloma and AA amyloidosis, our case highlights the importance of searching for an underlying monoclonal gammopathy like MM in a patient with a confirmed diagnosis of AA amyloidosis.

## Introduction

Amyloidosis is a rare group of disorders characterized by the extracellular deposition of fibrillar, misfolded, protein aggregates that are insoluble and thus interfere with the function of the tissue affected. It can be acquired or hereditary and can affect one specific organ (liver, intestines, spleen, kidney, heart, nerves, and blood vessels) or be systemic [1,2]. The further classification of systemic amyloidosis is based on the chemical analysis of the protein deposited, the most common types affecting humans being immunoglobulin-light-chain related amyloidosis or primary (AL), amyloid A or reactive/secondary amyloidosis (AA), amyloid transport protein transthyretin (ATTR), and dialysis-related amyloidosis (beta-2 microglobulin type) [3].

Monoclonal gammopathies, such as multiple myeloma (MM), monoclonal gammopathy of unknown significance (MGUS), and Waldenstrom macroglobulinemia, are usually associated with light chain (AL) amyloidosis, where there is light chain deposition in tissues in the setting of a plasma cell dyscrasia or neoplasia. In fact, approximately 10-15% of patients with AL amyloidosis will eventually be diagnosed with multiple myeloma. There are case reports of patients with MM who present with gastrointestinal symptoms and are later found to have amyloid deposits in gastrointestinal biopsies; pathology reports in all cases are read as AL amyloidosis [4].

We present a case of a patient with no previously diagnosed multiple myeloma who presented with gastrointestinal bleeding. Pathology from an intestinal sample showed AA amyloidosis and further investigation led to the finding of multiple myeloma. To our knowledge, this is the first case reported of multiple myeloma presenting with gastrointestinal bleeding where immunohistochemistry shows AA amyloidosis without evidence of systemic AA amyloidosis.

## Case presentation

A 57-year-old man, an everyday smoker, with a past medical history of colonic diverticulosis, presented to the emergency department complaining of right lower quadrant pain associated with melena. The patient reported dark red blood in stools with each bowel movement for one week, associated with fatigue and shortness of breath that worsened three days before admission. He denied non-steroidal anti-inflammatory drugs (NSAIDs) or blood thinner use. Laboratory workup showed macrocytic anemia with a hemoglobin level of 10 g/dL that dropped to 8.5 g/dL 12 hours later (his baseline hemoglobin was 14-15 g/dL one year before), platelets were 140 x10*3/uL, erythrocyte sedimentation rate (ESR) was elevated to 76 mm/hr and C-reactive protein (CRP), as well as renal function, were normal (Table 1).

**Table 1:** Laboratory results

Test	Value	Reference range
Erythrosedimentation rate, ESR	76	0-20 mm/hr
C-reactive protein, CRP	0.18	0.00-1.00 mg/dL
Antinuclear antibody, ANA	Negative	Negative
Lactate dehydrogenase, LDH	108 IU/L	100-190 IU/L
Immunofixation, serum	Immunoglobulin G (IgG) monoclonal protein with Kappa light chains IgG 3808	
Immunofixation, urine	IgG monoclonal protein with Kappa light chains	
Free Kappa light chains, urine	11290.98	1.17-86,46 mg/dL
Free light chains, serum	Free Kappa Lt chains 465.8	3.3-19.4 mg/dL
M spike, urine	65.5	Not observed
Protein electrophoresis, serum, SPEP	M spike 2.8 A/G ratio 0.7	0.4- 1.8 g/dL 0.7 -1.7
Anti-parietal cell antibodies	<1.0	0.0-20.0 units
Anti-intrinsic factor antibody	15.8	0.0- 1.1 AU7mL
Vitamin B12	160 pg/mL	232−1245 pg/mL
Hepatitis C	Non-reactive	Non-reactive
Hepatitis B	Immune	

**Figure 1:**
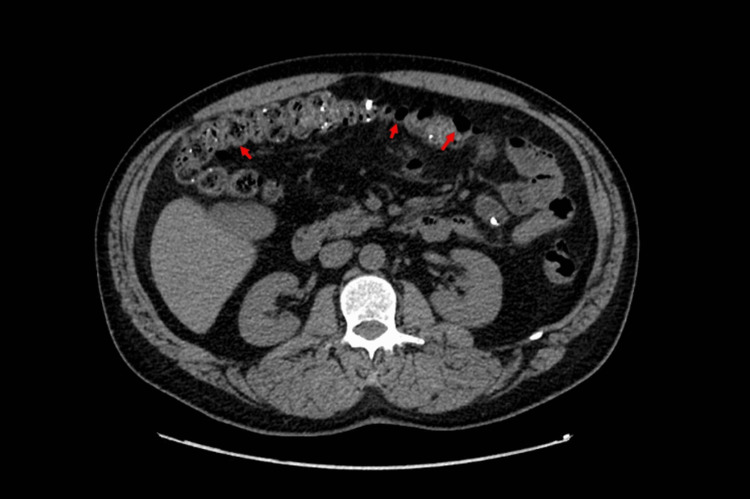
CT abdomen showing diverticulosis of the entire colon (red arrows)

**Figure 2:**
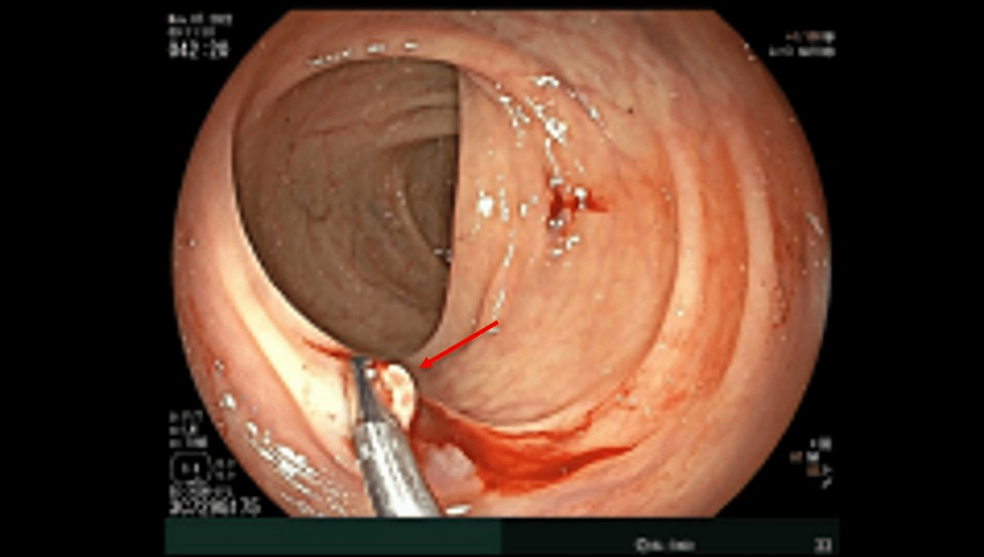
Solitary ulcer of the descending colon, clipped (red arrow)

**Figure 3:**
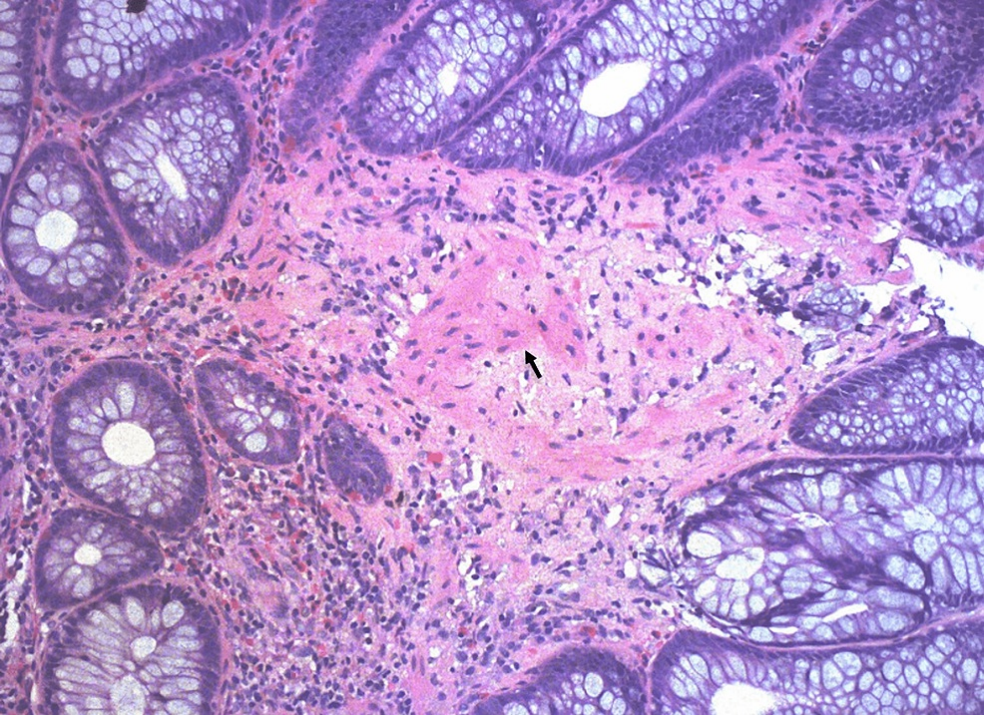
Eosinophilic amyloid deposition seen with hematoxylin and eosin stain of the colonic sample (20x, black arrow)

**Figure 4:**
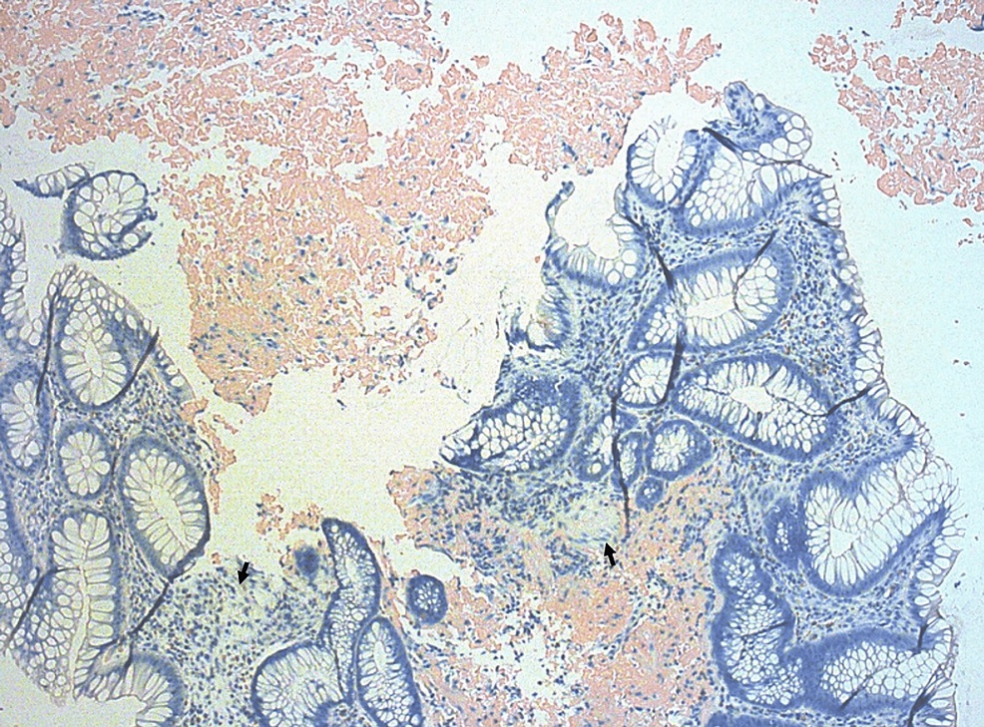
Congo red with pale pink staining and focal apple-green birefringence (black arrows) in polarized light microscopy (10x)

Physical exam was remarkable for diffuse abdominal pain and a small, one-centimeter, reducible umbilical hernia. Murphy's sign was negative, with no peritoneal signs. No palpable cervical, supraclavicular, or axillary adenopathy existed. On the digital rectal exam, there was no evidence of external hemorrhoids or gross blood. Computerized tomography angiography (CTA) of the chest, abdomen, and pelvis was positive for scattered diverticula throughout the colon (Figure 1). Echocardiogram revealed normal left ventricular wall motion with an ejection fraction of 65%. Further infectious and autoimmune workups, such as hepatitis panels, human immunodeficiency virus (HIV), syphilis, and nuclear antigen antibody (ANA), were all negative (Table 1). The patient underwent a colonoscopy for the source of bleeding, which showed severe diverticulosis in the entire examined colon with no evidence of active diverticular bleeding as well as a single (solitary) ulcer in the descending colon, which was clipped and biopsied (Figure 2). Upper endoscopy revealed gastric (antrum) and duodenal nonbleeding ulcers, pathology was positive for mild active chronic gastritis and numerous Helicobacter pylori. The patient was started on quadruplet therapy for four weeks with a proton pump inhibitor, tetracycline, metronidazole, and bismuth salt. The pathological anatomy of the colonic ulcer revealed non-dysplastic colonic mucosa with amorphous perivascular deposits in the lamina propria and loose detached fragments. Congo Red showed pale pink staining with focal apple-green birefringence in polarized light microscopy (Figures 3, 4); immunostaining for amyloid A (AA) protein was positive.

Looking for a primary diagnosis, serum protein electrophoresis (SPEP) was sent, which showed an M spike in the gamma region of 2.6 g/dL, free Kappa light chains of 465 with elevated free Kappa/lambda chains ratio of 113. Urine immunofixation (UIF) had IgG monoclonal protein with free Kappa light chains. Serum immunofixation showed elevated IgG monoclonal protein of 3808 with Kappa light chain specificity, and low immunoglobulin M (IgM) and IgA (<5) (Table 1). In light of these findings, a bone marrow biopsy was planned.

**Figure 5:**
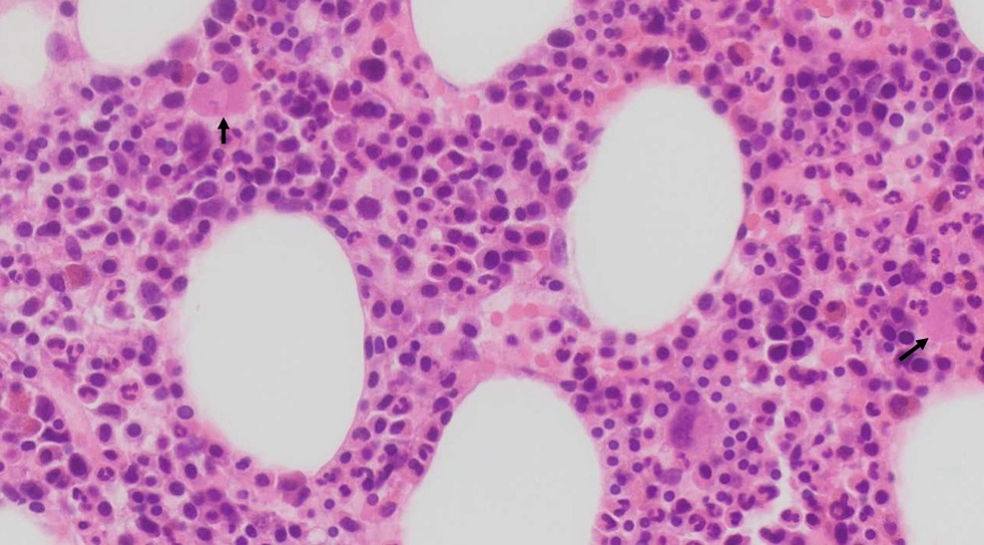
Bone marrow biopsy and aspirate with 5-15% increase in plasma cells (black arrows), diffusely scattered (20X)

**Figure 6:**
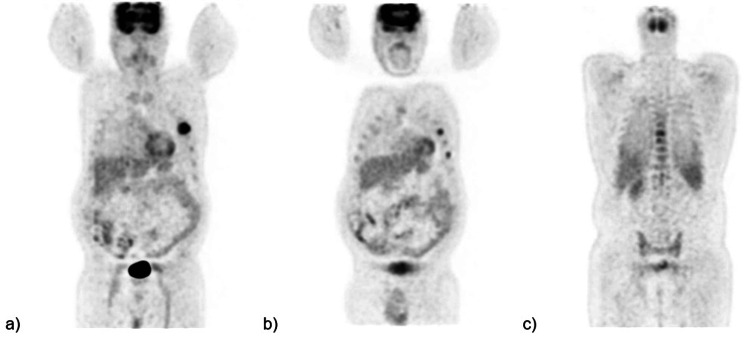
PET CT scan a, b) Left fifth and seventh rib fractures with increased metabolic activity. c) Vertebral lytic lesions at the T7, T8 levels. PET: positron emission tomography

Hemoglobin levels slowly went back up, no further bleeding episodes were documented, and the patient was discharged with gastroenterology and hematology follow-up. After discharge, a bone marrow biopsy was performed by the hematology department. The biopsy showed normocellular marrow with cellularity of 45-50%, trilineage hematopoiesis with maturation, and plasmacytosis of 5-10%, diffusely scattered (Figure 5). Concurrent flow cytometry was interpreted as IgG Kappa plasma cell myeloma (15% of total cells by flow cytometry). In the phenotype, a monoclonal plasma cell population (CD38 bright) represented 15% of the total cells. The neoplastic plasma cells expressed cytoplasmic IgG Kappa and CD117. CD20 was negative. B-cells were polyclonal (0.5% of total cells). There was no amyloid protein on the bone marrow sample. With the diagnosis of multiple myeloma, a positron emission tomography (PET) CT scan was ordered to rule out lytic lesions. The PET scan revealed left fifth and seventh rib fractures with surrounding soft tissue fullness and associated increased metabolic activity as well as vertebral lytic lesions at C5, T7, and T8, with the largest lytic lesion at T7 measuring up to 20 mm with increased metabolic activity (Figure 6).

An abdominal fat pad biopsy was performed to rule out systemic AA amyloidosis. On the fat tissue biopsy, there was no evidence of plasma cell neoplasm and Congo red stain was negative for amyloid deposition. 

The clinical picture and workup were consistent with multiple myeloma and the isolated AA amyloid deposits seemed more like an incidental finding, likely related to chronic inflammation. Daratumumab + weekly subcutaneous bortezomib, cyclophosphamide, and dexamethasone regimen was started. Revlimid was avoided, as the patient was a transplant candidate. Denosumab and calcium + vitamin D were also started for bone health and valacyclovir for herpes zoster virus (HZV) prophylaxis. The patient was also started on radiotherapy for spine and rib lesions.

## Discussion

Given the variable etiologies and clinical presentation of amyloidosis, it is extremely challenging to assess epidemiology. As per the National Institutes of Health (NIH), 78% of amyloidosis cases diagnosed every year in the United States are associated with AL amyloidosis, 10-20% are secondary to ATTR amyloidosis, and only 6% account for AA amyloidosis [3]. The incidence is higher in older people, with a mean age of 63, and it is more common in males [1,3].

The diagnosis of amyloidosis is made with a histological demonstration of amyloid deposition in a specific organ biopsy with a later confirmation of the amyloid subtype [2]. Amyloid typing is a crucial step for the correct management of the patient. It can be done through different mechanisms: immunohistochemistry (IHC), immunofluorescence (IF), and immunogold labeling [5]. There is no consensus regarding which method is the gold standard; however, the immunohistochemistry antibody panel to all different types of amyloid proteins has proven to be an accurate and standardized method [5].

Over 25 proteins have been identified to cause amyloidosis [2]. These amyloid protein precursors possess the characteristic of misfolding and causing aggregation, which is possible through different mechanisms: aging, making proteins more prone to misfolding, as in the case of transthyretin senile systemic amyloidosis; excessive high concentration of proteins in the serum such as in dialysis-related amyloidosis where beta-2M accumulates; replacement of amino acids on proteins making them more likely to misfold and aggregate such as in hereditary amyloidosis [3]. Tissues affected by amyloid protein suffer disruption of their normal architecture, and thus its function; the deposition of the misfolded protein also leads to an inflammatory reaction, oxidative stress, and apoptosis [2,3].

Serum amyloid (SSA), which is the protein deposited in AA amyloidosis, is an acute phase reactant. AA amyloidosis is always secondary to an underlying condition. It is associated with chronic inflammation, chronic infections, hereditary disorders, hematological disorders, and neoplasms [3,6]. Hematologic diseases that have been demonstrated to have a strong association with AA amyloidosis include neoplastic disorders such as Waldenström’s macroglobulinemia and non-Hodgkin’s and Hodgkin’s lymphoma. Multiple myeloma and MGUS, on the other hand, are recognized in the literature as having an unlikely association with AA amyloidosis [7]. This being said, when encountering a patient with such findings, a full workup needs to be performed to rule out other causes, confirm the diagnosis, and give appropriate treatment.

Multiple myeloma (MM) is a disorder characterized by the overproduction of monoclonal immunoglobulins (light or heavy) by plasma or B cells that infiltrate the bone marrow causing immunosuppression, nephropathy, neuropathy, and lytic bone lesions [7]. High levels of interleukin 6 (IL-6), which is an acute phase reactant, have been found in one-third of MM patients, and higher circulating levels are associated with poor prognosis of the disease. As explained by Terre et al., there is an ongoing discussion about the association of these cytokines with the production of inflammation leading to AA amyloid deposition [8].

As mentioned before, 15% of AL amyloidosis patients will develop MM and up to 30% of multiple myeloma patients will have evidence of subclinical amyloidosis deposits in different tissues, such as in bone marrow, fat pad biopsies, liver, intestines, and kidney biopsies [9]. Gastrointestinal (GI) amyloidosis has been described as the presence of GI symptoms with biopsy-proven amyloidosis, however, biopsy-positive GI amyloidosis is a rare finding [1,10].

In a literature review published in 2020, where 11 multiple myeloma cases reported from the year 2001 to 2016 were analyzed, the patients initially presented with gastrointestinal symptoms (hematemesis, hematochezia, macroglossia, chronic gastric ulcers, non-resolving small bowel obstruction). Biopsies were found to have amyloid deposits in different parts of the GI tract: stomach, duodenum, small bowel, colon, rectum, and liver [4]. In all cases, the pathology was reported as positive for AL amyloidosis, or only the finding of amyloidosis was reported without specifying the type of amyloid protein. We performed our own literature review (Table 2) using PubMed's advanced search builder and the words: gastrointestinal bleed, multiple myeloma, and amyloidosis with no filters. This search displayed 40 results, we included all cases with GI bleeding as a presentation where pathology was positive for amyloidosis and a diagnosis of multiple myeloma. Only nine cases were included in our review (reasons for exclusion were: baseline diagnosis of systemic amyloidosis in 29 cases, and in the other two cases, the presentation was not GI bleed). Of the nine cases, five were women and four were men, they all presented with gastrointestinal bleeding, with or without a previous diagnosis of multiple myeloma [11-19]. The GI bleed led to upper endoscopy and/or colonoscopy, where further GI pathology revealed amyloidosis. Of these, amyloid typing was reported only in one case [11]. In the other cases, the diagnosis of AL amyloidosis was assumed based on the baseline diagnosis of multiple myeloma or the location of amyloid deposits (mucosa and submucosa).

**Table 2:** Multiple myeloma presenting with GI bleeding and biopsy findings of amyloidosis without evidence of systemic amyloidosis MM: multiple myeloma; SMM: smoldering multiple myeloma

#	Author, year	Diagnosis of MM	GI symptom	GI biopsy site	Pathology report	Amyloid typing
1	Marques M, 2011 [12]	MM	Hematemesis	Gastric mucosa	Abundant deposits of eosinophilic and hyaline material. Congo red staining showing deposits of eosinophilic and hyaline material positive for an amyloid substance.	Not reported
2	Waleed, 2012 [13]	No previous diagnosis	Hematochezia	Rectal ulcer	Congo red revealed amyloid deposition within lamina propria and within the walls of blood vessels	Not reported
3	Cho, 2013 [14]	MM	Hematochezia	Transverse colon	Colonic biopsies stained with Congo red and viewed under polarized light revealed submucosal birefringent deposits characteristic of amyloidosis	Not reported
4	Gjeorgjievski et al, 2015 [11]	SMM	Melena	Mid-gastric body ulcer	Peptides were extracted from Congo red-positive areas after microdissection of paraffin-embedded stomach biopsy specimens	Mass spectrometry: AL- (lambda-) amyloidosis.
5	Rodriguez, 2016 [15]	MM	Melena	Proximal jejunum ulcer	Congo red histochemical staining of biopsy specimens demonstrated apple-green birefringence consistent with amyloid deposition	Not reported
6	Kim SY et al, 2016 [16]	No previous diagnosis	Dyspepsia and heartburn	Stomach (gastrectomy)	Amorphous pinkish hyaline deposits were observed in vessel walls. Congo red stain indicated a greenish birefringence corresponding to hyaline deposits.	Not reported
7	Singh et al, 2016 [17]	MM	Hematemesis and melena	Ulcer in the fundus and body of the stomach	Diffuse amyloid deposition in the fundus, distal body, and antrum of the stomach.	Not reported
8	Zhou H, 2018 [18]	No previous diagnosis	Hematemesis	Gastric antrum, ascending colon	Amyloid granules are visible below the epithelium in the gastric antrum and in the ascending colon. In both samples, granules were stained positive on Congo red staining.	Not reported
9	Chan, 2021 [19]	No previous diagnosis	Hematemesis, melena	Duodenal ulcer	Congo red staining revealed apple green birefringent material under polarized light, consistent with amyloid deposition	Not reported

Although the association of AA amyloidosis and MM is reported as unlikely, there is evidence of patients with a diagnosis of monoclonal gammopathies and associated AA amyloidosis. A retrospective review of cases with AA amyloidosis presenting with a diagnosis of monoclonal gammopathy was performed [8]. The review included all cases with confirmed diagnoses of AA amyloidosis through pathology and immunohistochemistry from 1946 to 2020 in three amyloidosis centers in France. Of all cases reviewed, monoclonal gammopathy was identified as the most likely cause of AA amyloidosis in 12 cases; out of this cohort, only one patient had multiple myeloma with amyloidosis found in the peripheral nervous system. Although a rare association, these findings propose that the inflammatory process of monoclonal gammopathies could lead to SSA accumulation in tissues [8]. This theory is also supported by the observation of a cure of AA amyloidosis after the patients were treated for the baseline disease such as in one of the cases of Waldenstrom macroglobulinemia (WM) where amyloid protein depositions disappeared after treatment of WM [20].

## Conclusions

Although there is little evidence in the literature of the association or even causative relationship between multiple myeloma and AA amyloidosis, our case highlights the importance of searching for an underlying monoclonal gammopathy like MM in a patient with a confirmed diagnosis of AA amyloidosis and vice versa. With this literature review, it has also been put in evidence the necessity of establishing the practice of completing the full workup of amyloidosis with proper identification of the amyloid protein deposited whenever there is a baseline diagnosis of MM, without evidence of systemic amyloidosis, instead of assuming that in all cases, the amyloid deposit found would be AL.
